# Recent Advances on MOF Derivatives for Non-Noble Metal Oxygen Electrocatalysts in Zinc-Air Batteries

**DOI:** 10.1007/s40820-021-00669-5

**Published:** 2021-06-07

**Authors:** Yuting Zhu, Kaihang Yue, Chenfeng Xia, Shahid Zaman, Huan Yang, Xianying Wang, Ya Yan, Bao Yu Xia

**Affiliations:** 1 School of Materials Science & Engineering, University of Shanghai for Science and Technology, 516 Jungong Road, Shanghai, 200093 People’s Republic of China; 2 Key Laboratory of Material Chemistry for Energy Conversion and Storage (Ministry of Education), Hubei Key Laboratory of Material Chemistry and Service Failure, Wuhan National Laboratory for Optoelectronics, School of Chemistry and Chemical Engineering, Huazhong University of Science and Technology (HUST), 1037 Luoyu Road, Wuhan, 430074 People’s Republic of China; 3 CAS Key Laboratory of Materials for Energy Conversion, Shanghai Institute of Ceramics, Chinese Academy of Sciences (SICCAS), Shanghai, 200050 People’s Republic of China

**Keywords:** Metal–organic framework, Non-noble metal, Oxygen electrocatalysts, Air electrode, Zinc-air batteries

## Abstract

Oxygen electrocatalysts are of great importance for the air electrode in zinc-air batteries (ZABs). Owing to the high specific surface area, controllable pore size and unsaturated metal active sites, metal–organic frameworks (MOFs) derivatives have been widely studied as oxygen electrocatalysts in ZABs. To date, many strategies have been developed to generate efficient oxygen electrocatalysts from MOFs for improving the performance of ZABs. In this review, the latest progress of the MOF-derived non-noble metal–oxygen electrocatalysts in ZABs is reviewed. The performance of these MOF-derived catalysts toward oxygen reduction, and oxygen evolution reactions is discussed based on the categories of metal-free carbon materials, single-atom catalysts, metal cluster/carbon composites and metal compound/carbon composites. Moreover, we provide a comprehensive overview on the design strategies of various MOF-derived non-noble metal–oxygen electrocatalysts and their structure-performance relationship. Finally, the challenges and perspectives are provided for further advancing the MOF-derived oxygen electrocatalysts in ZABs.

## Highlights

- This review summarizes the recent progress and application of different metal-organic frameworks (MOFs)-derived non-noble metal materials for zinc-air batteries in the past few years.
- This work gives extensive insights in understanding the relationship between design strategies and structure-activity relationship.
- The challenges and prospects of MOF-derived oxygen electrocatalysts for zinc-air batteries are proposed.

## Graphical abstract

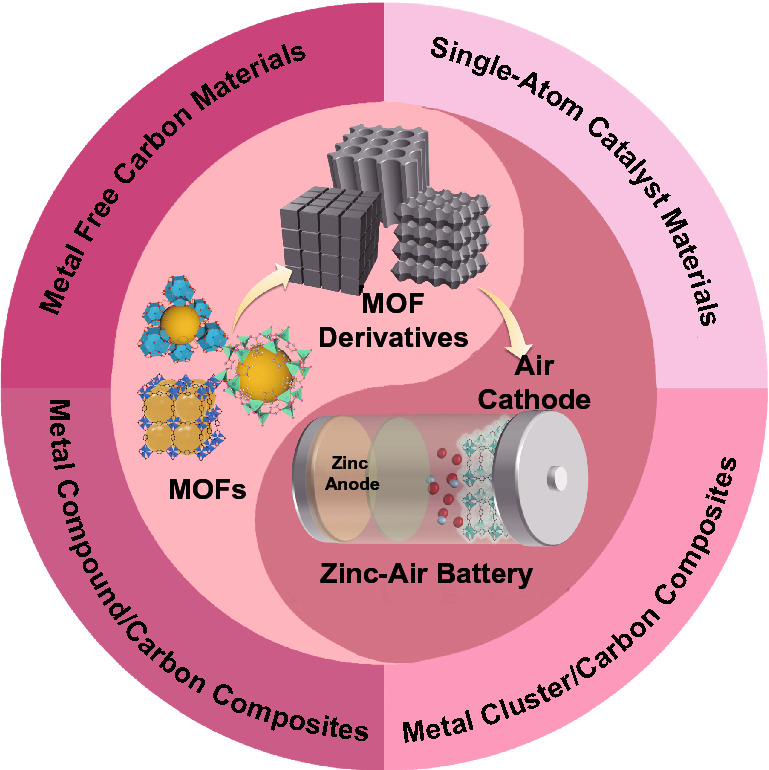

## Introduction

**Fig. 1:**
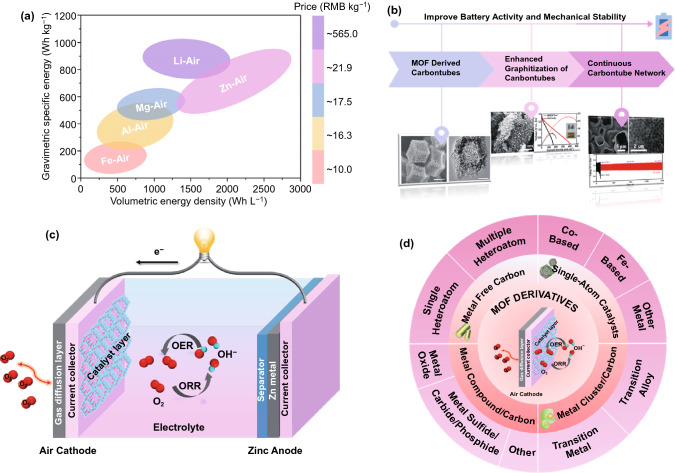
**a** Comparison of gravimetric specific energy and volumetric energy density of several batteries. **b** Progress of our research in the development of MOF derivatives in ZABs. **c** Typical device of the ZABs and air electrode of ZABs structure. **d** Classification diagram of MOF-derived catalysts for air cathode

Recent couple of decades has witnessed rapid technological development, which increased energy demands of the society; where the massive consumptions of conventional oil and gas resources have caused severe environmental problems [1]. Therefore, to eradicate the environmental issues, there is an urgent need to explore and develop new sustainable energy alternatives for the traditional non-renewable energy systems [2–4]. Hence, alternate electrochemical energy storage devices [5], such as fuel cells [6–11], lithium-ion batteries [12, 13], solar cells [14, 15], supercapacitors [16–18] and metal-air batteries [19–26] have been widely explored. Among these renewable sources, zinc-air batteries have attracted great attention due to their high specific energy density, environmental friendliness and safety [27–29]. Zinc-air batteries possess second highest weight-specific energy after the Li-air batteries, while the volume-specific energy is the highest among the other renewable energy sources (Fig. 1a). Thus, zinc-air batteries are considered to be a promising system because of their richly available raw materials, low cost, mildness of zinc electrode in the reaction process and the non-flammable aqueous electrolytes [30–32].

A typical zinc-air battery is usually composed of a zinc electrode, air electrode, electrolyte and a separator. The catalyst on the air electrode is one of the most important components, which is mainly responsible for the oxygen electrochemical reactions including oxygen reduction reaction (ORR) and oxygen evolution reaction (OER). The activity and stability of oxygen electrocatalysts play a significant role in the discharging-charging performance of ZABs [33–35]. At present, the most active catalysts are the precious metal-based materials, such as ruthenium and platinum-based catalysts. The high costs and low reserves of these precious metals greatly hinder their widespread applications in zinc-air batteries. Therefore, the development of highly efficient electrocatalysts based on non-noble metal materials for air electrodes has become imperative to realize the commercialization of ZABs.

Metal–organic frameworks (MOFs), also known as covalent organic frameworks (COFs) or coordination polymers, are crystalline porous materials with a periodic structure formed by the coordination of metal ions or clusters with organic ligands [36]. Up to now, MOFs have been applied to an ocean of fields, such as catalysis, energy storage, conversion, gas adsorption and separation [37]. Besides, as catalysts and catalytic host materials [38], MOFs are frequently used as a precursor to developing MOF-derived electrode catalysts, which not only retain the advantages of the original structure of MOFs but also provide enhanced conductivity and stability [39, 40]. Based on the versatile and unique structure–activity characteristics of MOF-derived nanomaterials toward oxygen electrocatalysis in ZABs, our group has contributed many leading, innovative and systematic works (Fig. 1b). We have reported a well-defined hollow structure composed of cobalt incorporated into nitrogen-doped carbon nanotubes by the thermal annealing of zeolitic-imidazolate frameworks (ZIFs) for the first time as electrocatalyst for ZABs [41]. This work provides a prospect for the development of highly active oxygen electrocatalysts in electrochemical energy devices [42–44]. To further enhance the activity and stability of MOF-derived carbon nanotubes for zinc-air batteries, the graphitization degree of carbon nanotubes can be improved from the enhanced carbon bonding at the micro–macro scale, to achieve the enhanced corrosion resistance, stability and conductivity of the electrocatalysts [45]. Thereafter, we focused on the design of both efficient and stable bifunctional oxygen electrocatalysts for long-life metal-air batteries [46–48]. A template method has been developed to prepare a variety of three-dimensional continuous carbon nanotube network materials for liquid and flexible solid-state zinc-air batteries to improve performance and cycle stability [49–51]. When applied to the rechargeable zinc-air batteries, strong cycle stability has been attempted up to 1600 h. Therefore, with substantial contributions in the preparation of MOFs derived oxygen catalysts for zinc-air batteries, we anticipate to work on the design of MOF-derived carbon-based catalysts, including optimizing the structure of carbon-based catalysts, improving the utilization of active metal atoms, improving the conductivity, stability, corrosion resistance of catalyst carriers, etc., to improve energy power density and cycling durability of ZABs.

There are many pieces of research on MOFs-based materials [52–54], however, most of them focus on the synthesis of MOFs-based catalysts for ORR or OER under different conditions, and little attention has been paid to the MOF-derived electrocatalysts as air cathode in ZABs, including ORR catalysts and OER/ORR bifunctional catalysts. Moreover, there is huge room to describe the relationship between catalyst material structures, oxygen electrocatalytic activity and the practical problems in the application of zinc-air batteries. In this review, the recent progress in MOF-derived oxygen electrocatalysts as air cathode in zinc-air batteries is comprehensively discussed and summarized with the focus on understanding the design strategies and the structure–activity relationship. Starting with a brief introduction to the fundamentals of oxygen electrolysis in ZABs, the recent advances on MOF-derived non-noble metal–oxygen electrocatalysts are successively reviewed for ORR and OER from the category of metal-free carbon materials, single-atom catalysts, transitional metal cluster/carbon composites and metal compound/carbon composites. In particular, these MOF-derived non-noble metal–oxygen electrocatalysts in the ZABs are reviewed based on the structure-performance relationship. Finally, the challenges and prospects of MOF-derived non-noble-metal oxygen electrocatalysts in ZABs are proposed. We hope that this review and the provided references will play a guiding role to contribute in the development of MOF-derived oxygen electrocatalytic materials in ZABs.

## Oxygen Electrolysis in ZABs

### Architecture and Working Mechanism

A typical configuration of zinc-air battery is usually composed of air cathode, zinc anode, electrolyte and separator (Fig. 1c) [55, 56]. A multitude of the anode material of ZABs usually uses a gel mixture mixed with granular zinc powder or a pure Zn plate. The air electrode is the key technology of ZABs. The oxygen in the air is the active and inexhaustible substance of electrode reaction, which makes the air electrode have high specific energy compared with the oxide electrode as a cathode material of general batteries. Generally, the gas diffusion layer, the current collector and the catalyst layer form a sandwich structure, which composes of the traditional air electrode (Fig. 1c). The components of the air electrode include: (1) current collector, the current collector is nickel mesh made of nickel metal, nickel foam or a cheaper metal mesh with nickel coating [31]. (2) catalytic layer, this is the place where ORR occurs in the primary ZABs, or OER and ORR occur in the secondary ZABs, which is the key to the performance of the ZABs, (3) gas diffusion layer, the main function of the gas diffusion layer is to let the reaction gas pass through smoothly and transport the corresponding gas needed for the reaction active layer. At the same time, the gas diffusion layer must prevent the gas diffusion channel from being covered due to the flow of electrolyte, which requires the gas diffusion layer to have a highly limited surface area. For example, polytetrafluoroethylene (PTFE) is generally the main part of the gas diffusion layer and can be doped with other carbon materials to form the gas diffusion layer [57].

Usually, the well-known working mechanism of ZABs is the oxidation–reduction reaction between zinc anode and air cathode during the discharge and charge process [27]. During the discharge process, oxygen diffuses to the air electrode, where it is reduced to hydroxyl ions under the action of an active catalyst. The hydroxyl ions generated on the air cathode migrate to the zinc anode through the separator, and then combine with zinc ions to form soluble zincate ion (Zn(OH)_4_^2−^), when the zincate ion in the electrolyte reaches saturation, it will decompose to ZnO. In this process, the electrons released by the reaction between zinc and hydroxide are transferred to the air cathode through an external circuit, and the oxygen in the air in contact with the air cathode undergoes an ORR at the air cathode [58]. During the charging process, OER occurs at the air cathode, and the final reaction is the decomposition of ZnO into Zn and O_2_. The specific process is:

$${\text{Air}}\;{\text{cathode}}{:}\quad {\text{O}}_{2} + 2{\text{H}}_{2} {\text{O}} + 4{\text{e}}^{ - } \to \, 4{\text{OH}}^{ - }$$

$${\text{Zinc}}\;{\text{anode}}{:}\quad {\text{ Zn}} \to {\text{Zn}}^{2 + } + 2{\text{e}}^{ - }$$

$${\text{Zn}}^{2 + } + 2{\text{OH}}^{ - } \to {\text{ Zn}}({\text{OH}})_{2}$$

$${\text{Zn}}({\text{OH}})_{2} \to {\text{ZnO}} + {\text{ H}}_{2} {\text{O}}$$

$${\text{Total}}\;{\text{reaction}}{:}\quad 2{\text{Zn}} + {\text{O}}_{2} \to \, 2{\text{ZnO}}$$

The catalysts employed in the air electrode determine the efficiency of the ZABs, therefore, reasonable assessment should be established to evaluate the oxygen electrochemical catalysts from a single reaction to the whole battery architecture. Generally, the performance of the ORR catalyst is evaluated by comparing the reduction peak potential, onset potential, half-wave potential and stability, while the performance of the OER catalysts is usually estimated by the overpotential to achieve the current density of 10 mA cm^−2^ as well as its durability. During the charge–discharge process of ZABs, the OER and ORR ﻿﻿occur on air electrode alternately. The challenge for the air electrode is the two different overpotentials needed to trigger the OER and ORR processes because the OER requires a larger overpotential to occur (≈2.0 V or even higher), but at this voltage, the ORR catalyst will be deactivated during high-voltage charging. Therefore, the catalysts with good ORR activity generally show poor OER performance. For this reason, the development of non-precious metal materials with both OER and ORR functional catalytic properties is crucial for the development of ZABs, especially rechargeable ZABs [59]. Besides, 6 M KOH is frequently used as the electrolyte for the primary batteries, and Zn(AC)_2_ is usually added to the 6 M KOH electrolyte in the rechargeable batteries. To assess the performance of the constructed ZABs, attention should be paid to open-circuit voltage (OCV), charging voltage (V_C_), discharging voltage (V_D_), peak power density (PPD), specific capacity, stability and other physical parameters. In light of the structure–activity relationship, the obtained performance results can be further used to optimize the design of the oxygen catalysts to achieve higher performance of ZABs [60].

### Oxygen Electrochemical Reactions in Air Electrode

It is obvious that for both primary ZABs and rechargeable ZABs, the ORR is an essential electrochemical process, which is the key reaction of the ZABs. The ORR mechanism is also complicated due to the multiple-electron reaction process. At present, the normally accepted ORR mechanism involves a four-electron reaction mechanism and/or two-electron reaction mechanism [61]. In the ORR, the best-performing precious metal catalysts are mainly four-electron reactions. The process of both reaction mechanisms is explained as follows:

Four-electron reaction:

$${\text{Acidic}}\;{\text{electrolyte}}{:}\quad {\text{O}}_{2} + 4{\text{H}}^{ + } + \, 4{\text{e}}^{ - } \to 2{\text{H}}_{2} {\text{O}}$$

$${\text{Alkaline}}\;{\text{electrolyte}}{:}\quad {\text{O}}_{2} + 2{\text{H}}_{2} {\text{O}} + 4{\text{e}}^{ - } \to 4{\text{OH}}^{ - }$$

Two-electron reaction:

$${\text{Acidic}}\;{\text{electrolyte}}{:}\quad {\text{O}}_{2} + 2{\text{H}}^{ + } + 2{\text{e}}^{ - } \to {\text{H}}_{2} {\text{O}}_{2}$$

$${\text{H}}_{2} {\text{O}}_{2} + 2{\text{H}}^{ + } + 2{\text{e}}^{ - } \to 2{\text{H}}_{2} {\text{O}}$$

$${\text{Alkaline}}\;{\text{electrolyte}}{:}\quad {\text{O}}_{2} + 2{\text{H}}_{2} {\text{O}} + 4{\text{e}}^{ - } \to {\text{HO}}_{2}^{ - } + {\text{OH}}^{ - }$$

$${\text{HO}}_{2}^{ - } + {\text{H}}_{2} {\text{O}} + 2{\text{e}}^{ - } \to 3{\text{OH}}^{ - }$$

In the rechargeable ZABs, the oxygen electrocatalyst is also vital to catalyze the reverse reaction of ORR, that is OER [62–66]. When charging, the zinc compound will be formed at the anode, and the air cathode will release oxygen [67]. Analogous to ORR, OER also involves the transfer of four electrons, so its reaction mechanism is also very complicated. At present, there are two main points of view to explain the reaction mechanism of OER, namely: (1) directly combine two M–O to generate O_2_; (2) first generate M-OOH, and then generate O_2_ to from M-OOH, here M means active site. Compared with the ORR, the OER kinetics of rechargeable ZABs are much slower, a charging voltage of about 2.0 V or higher is often required, and the open-circuit voltage of the discharging process is generally around 1.2 V. Furthermore, the excessive charging voltage will cause the corrosion of air electrode and oxidation of the electrocatalyst [29]. Certainly, the existing three-electrode structure allows a rechargeable zinc-air battery to be assembled using two single functional oxygen electrocatalysts to achieve dual functions. The three-electrode system battery increases the volume and weight of the battery while improving the stability, which inevitably reduces the volume and mass-energy. So, the air cathode catalyst is preferred to be functional for both OER and ORR instead of the three-electrode system. This puts forward higher requirements for the development of highly active and stable OER catalysts, even more, efficient bifunctional catalysts for catalyzing both OER and ORR.

In general, the charge–discharge process of ZABs requires two oxygen electrochemical processes, OER and ORR, on the air cathode. However, due to intrinsic high kinetic barrier of OER and ORR, highly efficient electrocatalysts are required to meet the desired power output. Considering the diverse structural characteristics of MOFs derived electrocatalytic materials, the MOF-derived oxygen electrocatalysts provide more possibilities for performance optimization in ZABs. Particularly, the high specific surface area can lead to more accessible active sites in contact with electrolyte. When MOFs is used as a support matrix, its crystal framework can ensure the uniform distribution of active units, which avoids the formation of large agglomerates and provides a diversified platform for the synthesis of high-performance metals or carbon-based derivatives [68–70]. Therefore, MOFs and their derivatives are promising electrode materials for ZABs.

## MOF-Derived Oxygen Electrocatalysts as Air Electrode in ZABs

MOFs are the organic–inorganic hybrid materials, which possess a variety of metal ions and organic ligands coordinated to form a variety of structures. Compared with the pristine MOFs, MOF-derived materials inherit the porous characteristics of original MOFs to a great extent, which realize the precise regulation of the active ingredients in the derived materials. We summarize the different types of MOF derivatives as oxygen electrocatalysts in ZABs (Fig. 1d). The following parts will review and discuss these advanced MOF-derived oxygen electrocatalysts successively from the category of metal-free carbon materials, single-atom catalysts, metal cluster/carbon composites and metal compound/carbon composites.

### MOF-Derived Metal-Free Carbon Materials

Carbon materials have the advantages of low cost, environmental acceptability, positive conductivity and stability [71, 72]. When doped with heteroatom such as nitrogen, phosphorus, boron, sulfur, oxygen and other heteroatoms, they often exhibit enhanced catalytic activity. Compared with metal-based catalysts, MOF-derived metal-free carbon materials are promising alternative electrocatalysts for ORR and are frequently employed as air cathode catalysts in ZABs [73–76].

**Fig. 2:**
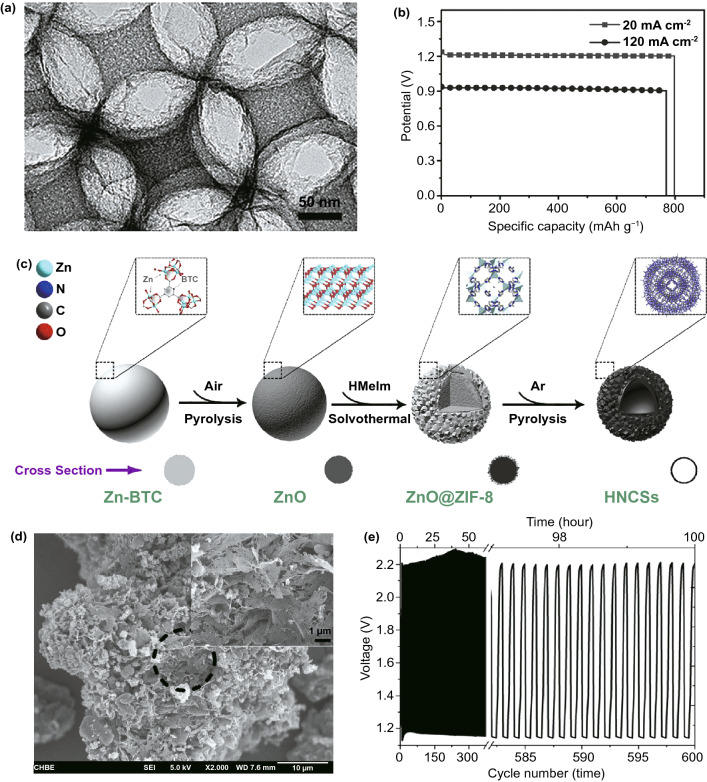
**a** TEM images and **b** specific capacity for ZABs of MOF-derived nitrogen-rich carbon photonic crystal (BHPC-950). Copyright © 2018 WILEY-VCH Verlag GmbH & Co. KGaA, Weinheim. **c** Schematic preparation of hollow N-doped carbon spheres through in-situ growth and calcination strategy. Copyright © 2019 Elsevier Ltd. **d** SEM image of BNPC-1100 and **e** cycling performance of the rechargeable Zn-air batteries using BNPC-1100 as the air cathode. Copyright © 2016 Elsevier Ltd

As a type of host materials, metal-free carbon materials derived from MOFs have also been studied as oxygen catalysts, especially the nitrogen-rich porous carbon (NPC) [77–79]. When used as cathode electrocatalysts in the ZABs, the doped N can terminate the electrical neutrality of adjacent carbon atoms and form a positively charged position due to the electron-withdrawing characteristics of N atoms. This character can promote oxygen adsorption and thus improve the cathode ORR performance. In a novel work by Yang et al. [80], an effective and universal silica-template strategy has been used to form an ordered macro-porous carbon skeleton with narrow connections/walls between the spherical voids (BHPC, Fig. 2a) by in-situ growing ZIF-8 crystal particles on the surface of the silica microspheres and subsequent carbonization to remove the template. Compared with NC-950 (1331 m^2^ g^−1^, 1.71 cm^3^ g^−1^), BHPC-950 had a larger total pore volume of 13.42 cm^3^ g^−1^, a larger specific surface area of 2546 cm^2^ g^−1^, high N content of 7.6 at% and ordered interconnected pore network, which highlighted the advantages of this dual template strategy. As shown in Fig. 2b, by using the BHPC-950 as an air electrode, the assembled ZABs could not only work at a high rate of 120 mA cm^−2^ but also provide an excellent capacity of 770 mAh g^−1^. Besides, two constructed ZABs connected in series could light up a light-emitting diode composed of 30 LEDs (2.2 V) for 12 h without brightness attenuation. These results indicated that the highly exposed graphite N under the double template had unique texture characteristics, which would promote the cathodic ORR in ZABs and could be used as a promising substitute for Pt-based catalysts in energy devices. Also, ZnO and ZIF-8 composite were used as the sacrifice template to fabricate hollow N-doped carbon microspheres (Fig. 2c) [81]. The large pore volume and high surface area in the microspheres not only promote the diffusion of electrolyte and gas molecules but also offer richly accessible active sites. Moreover, the addition of glucose as extra carbon source to the ZIF could improve the graphitization degree of samples and help to remove zinc metal and zinc compound impurities, giving an effective route to synthesize metal-free nitrogen-doped porous carbon [82]. These ZIF-derived metal-free electrocatalysts usually exhibit excellent electrocatalytic activity and operational stability for ORR with a great potential in ZABs. Therefore, as an advanced platform, MOF-derived porous carbon will significantly broaden the family of nanoporous carbon materials with novel structure and versatile properties for oxygen electrolysis in ZABs.

Multi-nonmetallic heteroatom doping is another important strategy to utilize the mutual synergistic effect of different atoms to improve the oxygen electrocatalytic performance of the carbon materials [83, 84]. Qian et al. [85] reported B-N double-doped porous carbon (BNPC) for ORR/OER catalysis by thermal decomposition of Zn-MOF (MC-BIF-1S) in an H_2_–Ar mixed atmosphere, which decomposed into cracked BNPC solids with a porous structure (Fig. 2d). It was a good example that MOF material was used to prepare the metal-free bifunctional electrocatalyst. The larger pores in the catalyst could greatly reduce the mass transfer resistance. The cracking and porous structure of BNPC produced a sea of large pores, which could be used as channels for reactants to pass through the electrode layer, thereby improving catalytic performance. BNPC-1100 as an oxygen catalyst on the air cathode in rechargeable ZABs exhibited a charge potential of 2.19 V at a current density of 2 mA cm^−2^, and the discharge potential was 2.16 V. The cycling stability of the assembled batteries was tested for 100 h without significant performance loss (Fig. 2e). Besides, S and N co-doped porous carbon was also synthesized by using urea as nitrogen source dimethyl sulfoxide as sulfur source in the MOF-5 template [86]. The synergistic effect of N and S in NS(3:1)-CMOF-5 as a metal-free electrocatalyst for ORR showed the highest initial potential, even comparable to the Pt/C catalyst. These porous carbon materials derived from self-sacrificial MOFs templates have more unique structural characteristics than traditional carbon materials. Inheriting the advantages of MOFs, the pore structure becomes more variable to meet the demand, and the specific surface area is greatly improved, which will affect the final performance of the material. Therefore, by selecting desired dopants and proper MOF precursors, multi-nonmetallic heteroatom-doped carbon materials can be ingeniously designed as advanced oxygen electrocatalysts, which are very promising to be used as catalytic materials for air cathode in ZABs.

### MOF-Derived Single-Atom Catalysts

Platinum/iridium-based catalysts are often benchmark electrocatalysts used to improve oxygen electrolysis in ZABs [87, 88], reducing the catalyst loading and increasing intrinsic catalytic activity of these noble metal-based oxygen electrocatalysts are critical to realizing their application on large-scale. In this regard, single-atomic catalysts (SACs) are promising due to favorable active sites uniformity, high product selectivity, multiple support types, high atomic efficiency and low precious metal usage [89–91]. However, it is difficult and challenging to synthesize SACs because with the dispersion of catalysts at the atomic level, the surface free energy increase sharply leading to serious aggregation [92]. Owing to the unique structural characteristics, MOFs present an effective a template or host to design SACs [93]. Besides, the MOF-derived noble metal SACs, reports on MOF-derived non-precious metal containing SACs are also widely explored [94–99]. The following parts will focus on the application of MOF-derived transition metal (e.g., Co, Fe and Mn) single-atom oxygen catalysts in ZABs.

#### MOF-Derived Co-based SACs

The nitrogen coordinated cobalt atom in the carbon matrix (Co–N–C) is considered to be the ideal material to replace the noble metal Pt-based materials for the ORR [100]. To improve the utilization, efficiency of active Co metal by designing the Co single-atom decorated N-doped porous carbon materials is an effective way [101, 102]. For instance, Zang et al. [103] used a Co-MOF to prepare the CoSA electrocatalyst in N-doped porous carbon nanosheets array by carbonization and acidification process. The obtained NC-CoSA in a wafer array had well-dispersed Co single-atoms, connected to the carbon network through Co–N bonds, because of the extra porosity in the carbon network and an active surface area generated by removing Co metal clusters. With Co nanoparticles grown on NC comparison, the NC-CoSA electrocatalyst with a high-density of Co–N_*x*_ active centers had the lower OER overpotential and higher ORR saturation current, which indicated that Co metal clusters were driving OER, and ORR aspect is superfluous. Therefore, the NC-CoSA electrocatalyst assembled on the carbon cloth used as the air cathode without binder, and additive was applied in the solid-state ZABs. The constructed batteries demonstrated positive cycle stability and high open-circuit potential. Unlike the simple carbonization and acidification method, Ji et al. [104] reported a novel impregnation carbonization acidification (ICA) method to prepare atom-dispersed Co–N sites within the porous carbon sheet arrays grown on carbon nanofibers using ZIFs and electrospun nanofibers (ENFs) as precursors. During the synthesis process, ZIF-Ls evolved into N-doped carbon flakes, while cobalt ion nitrogen coordination units in ZIFs were reduced in-situ to form atom-dispersed Co–N sites. The excess cobalt atoms aggregated and caused the formation of Co clusters, which were removed by an acid leaching process to form CoSA@NCF/CNF with outstanding flexibility. Therefore, the wearable ZABs composed of the CoSA@NCF/CNF air electrode had a high deformation tolerance and promising potential as an integrated batteries system.

**Fig. 3:**
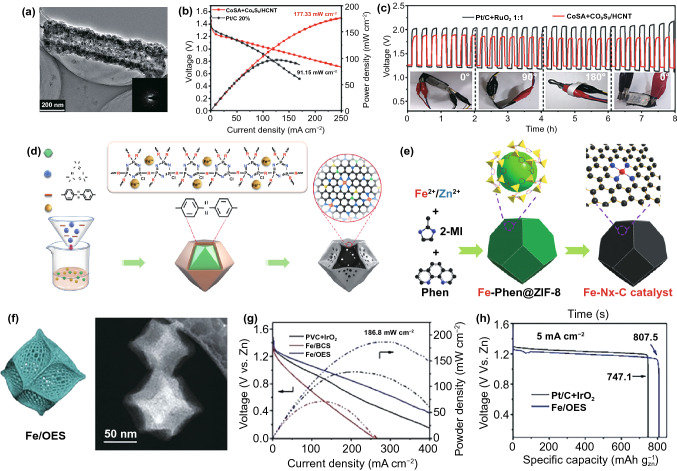
**a**–**c** TEM image, power density curves and discharge–charge cycling profiles (solid-state) of Zn-air batteries based on CoSA + Co_9_S_8_/HCNT. Copyright © 2020 WILEY–VCH Verlag GmbH & Co. KGaA, Weinheim. **d** Preparation of single iron atomic sites supported on nitrogen, phosphorus and sulfur co-doped hollow carbon polyhedron (Fe-SAs/NPS-HC). Copyright © 2018 Nature Publishing Group. **e** Synthesis diagram of Fe–N_*x*_–C catalyst. Copyright © 2019 WILEY-VCH Verlag GmbH & Co. KGaA, Weinheim. **f**–**h** SEM image, power density plots and the specific capacities of Fe/OES. Copyright © 2020 Wiley‐VCH Verlag GmbH & Co. KGaA, Weinheim

In addition to above discussed single CoSACs, MOF-derived Co-monatomic catalyst complexes also display excellent electrocatalytic properties. A representative example is hollow carbon nanotubes integrated with single Co atoms and Co_9_S_8_ nanoparticles (CoSA + Co_9_S_8_/HCNT) prepared by the in-situ self-sacrificing method [105]. A ZnS/ZIF-67 hybrid was used as a template, after the carbonization treatment, the ZnS self-sacrificing nanorods are formed a tubular structure, which served as a sulfur source for the formation of Co_9_S_8_ nanoparticles (Fig. 3a). The obtained CoSA + Co_9_S_8_/HCNT showed outstanding oxygen electrocatalytic activity, and its potential difference (the difference between *E*_*j*=10_ and *E*_1/2_) was 0.705 V, much smaller than the potential difference of Pt/C + RuO_2_ (0.777 V). The peak power density of the liquid ZABs assembled with CoSA + Co_9_S_8_/HCNT reached 177.33 mW cm^−2^, much better than Pt/C + RuO_2_ (Fig. 3b). And at 1 mA cm^−2^, the flexible Zn-air batteries based on CoSA + Co_9_S_8_/HCNT catalyst still maintained a stable discharge/charge potential after a long cycle and had excellent durability (Fig. 3c).

#### MOF-Derived Fe-Based SACs

In parallel, various MOF-derived Fe-based single-atom catalysts are also widely studied. Previously, Wang and his coworkers reported a hollow structure composed of single iron atom sites on N, P and S co-doped hollow carbon polyhedron (Fe-SAs/NPS-HC) constructed by MOF@polymer composite [106]. They highlighted that the polymer-based coating modulated the electrons of the active metal center through the Kirkendall effect while the close coordination of N and the long-distance interaction of S and P promoted the construction of hollow structures (Fig. 3d). Thus, the as-prepared Fe SAs/NPS-HC catalyst showed an excellent ORR performance in alkaline medium, with a half-wave potential (*E*_1/2_) of 0.912 V, while in acidic media, a *E*_1/2_ of 0.791 V was obtained, which was close to the catalytic performance of Pt/C catalyst and better than the majority of non-noble metal catalysts. Density functional theory (DFT) calculations revealed that the dispersion of N-coordinated Fe atoms and the electronic effects of surrounding S and P atoms are responsible for the efficient and satisfactory dynamics of Fe-SAs/NP-HC. The atomic Fe center provided electrons, which weakened the charge of Fe (Fe^δ+^), thereby weakening the binding of Fe (Fe^δ+^) charge to OH species. Moreover, the constructed ZABs indicated that Fe-SAs/NPS-HC had strong competitiveness compared with Pt/C, suggesting the prospects in energy storage and conversion devices.

In these MOF-derived Fe-based SACs, Fe–N_6_, Fe–N_4_ and Fe–N_2_ coordination are often considered to be the main active components that affecting the oxygen electrocatalytic performance [107–109]. Fe–N–C is one of the most representative MOF-derived transition metal single-atom electrocatalyst material. Combination of experiments and DFT calculations, Han et al. [110] systematically studied the proximity effect of two adjacent Fe–N–C sites on ORR in a monodispersed Fe–N–C single-atom catalyst, which is essential for a more comprehensive understanding of how the single-atom catalyst works. To further enhance the oxygen electrochemical process of the Fe–N active sites in ZABs, Sun’s team [111] used ZIF-8 to synthesize single-atom Fe–N_*x*_–C electrocatalyst by in-situ incorporating Fe^2+^ ions coordinated with 1,10-phenan-throline complexes (Fe-Phen) into the nanocages during the growth of ZIF-8 followed by pyrolysis (Fig. 3e). Owing to the Fe-Phen species providing both Fe^2+^ and organic ligands (Phen), the Fe-Phen@ZIF-8 precursor played an important role in the preparation of single-atom catalysts. After the pyrolysis at 900 °C under argon atmosphere, the precursor was converted into the isolated Fe single-atom on nitrogen-doped carbon frameworks, which allowed the iron atoms to be more dispersed to obtain single-atom to increase the active sites. The obtained Fe–N_*x*_–C exhibited a higher half-wave potential of 0.91 V versus RHE than that of commercial Pt/C (0.82 V). As a cathode catalyst in the primary ZABs, it exhibited an excellent electrochemical performance at a high open-circuit voltage of 1.51 V. In rechargeable ZABs, it showed marvelous cycling performance within 300 h with an initial round-trip efficiency of 59.6%. In addition, all-solid-state ZABs with Fe–N_*x*_–C catalyst show 1.49 V and the cycle life was up to 120 h.

Besides, modifying the Fe-based single-atom with appropriate MOFs, engineered morphology of the oxygen catalyst is equally conducive to optimize the ZABs performance by enhancing the mass diffusion and electron transfer between the catalyst layers, thereby increasing the utilization of active centers. As a proof-of-concept study, Hou et al. designed an overhang structure that modified an isolated monoatomic iron site by a silica-mediated MOF template method for ORR (Fig. 3f) [112]. It was found that SiO_2_ coated MOFs could generate outward adsorption force, causing the anisotropic thermal contraction of the MOF precursor. The dodecahedron plane collapsed during the pyrolysis process, and the fringe of ZIF-8 was maintained. At the same time, the obtained N-doped carbon could reduce Fe^3+^ ions and combine with adjacent N/C atoms to form Fe–N_4_–C sites. The as-prepared oxygen catalyst showed good performance in the ZABs, reaching a capacity of 807.5 mAh g^−1^, a high peak power density of 186.8 mW cm^−2^ and a decent energy density of 962.7 Wh kg^−1^, which is comparable to the Pt/C catalyst (Fig. 3g, h). This superior activity of the MOF-derived Fe SACs was contributed by the rich edge structure, effective three-phase boundaries, which enhanced the mass transport of reactants to accessible monoatomic iron sites. All the above works prove the advantage and practicability of MOFs as platform materials for synthesis effective Fe-based SACs as oxygen electrocatalysts for the ZABs.

#### MOF-Derived Mn-Based SACs

In addition to Co-based and Fe-based single-atom catalysts, many other single-atom catalysts have also attracted great attention, such as Mn-based catalysts. With the assistance of theoretical calculation, Lin et al. [113] found that the local coordination environment could adjust its intrinsic catalytic activity by changing the electronic structure of the Mn center. Furthermore, the high activity of the pyridine-N-coordination Mn configuration was found to originate from the moderate adsorption strength of the ORR intermediate and the reasonable position of the d-band center, which promoted the ORR process. To prove such theoretical results, they successfully prepared Mn-SA electrocatalysts composed of atomically dispersed pyridine-N coordination Mn atoms in the carbon skeleton. The electrochemical tests showed that the Mn-based SACs had an excellent performance on ORR showing promising half-wave potential of 0.87 V and diffusion current-limiting performance of ZABs under alkaline conditions, which was superior to most Mn-based nanomaterial catalysts and Pt/C catalysts. More recently, well-dispersed Mn single-atoms anchored nitrogen-doped carbon with Mn–N_4_ configuration catalyst (Mn-SAS/CN) was prepared by one-step thermal activation of Mn(CH_3_COO)_2_@ZIF-8 precursor [114]. The operando X-ray absorption spectroscopy analysis showed that the active sites of Mn changed with the applied potential under the basic ORR condition, and Mn^L+^–N_4_ without covering OH_ads_ was the catalytic center. Further DFT calculations showed that the excellent ORR performance is attributed to the easier electron transfer from Mn^L+^–N_4_ site to the adsorbed *OH species. The preparation of single-atom materials is different from other materials, and the special structure of MOFs makes the probability of uniform dispersion of single-atom. Impressively, the Mn-N_4_ oxygen electrocatalysis material demonstrated high peak power density and excellent durability when assembled in ZABs, much higher than the bulk of Fe- and Co-based SACs and the commercial Pt/C, showing great potential to replace Pt in practical energy devices.

### MOF-Derived Metal Cluster/Carbon Composites

Although MOFs derived metal-free carbon materials possess better structural advantages than common carbon materials, the main problem of metal-free carbon materials is the lack of metal catalytic centers, which makes their reaction kinetics relatively slow due to the low activity of catalytic sites. Moreover, the MOFs derived metal-free materials are required to remove the metal elements within the electrocatalysts, making the synthesis process complicated and expensive. As for MOF-derived single-atom catalysts mentioned earlier, it is reported the surface area of monatomic metals increases significantly in single-atom catalyst, which leads to the sharp increase of surface free energy. Therefore, a large number of metal atoms are prone to agglomerate and inevitably lead to the deactivation of the catalyst during the oxygen electrolysis process [115]. Also, the limited loading of single-atom catalyst makes it far from being able to meet the practical application. Whereas MOF-derived carbon composites, doping with transition metal clusters present a feasible and promising way for the strong binding force between carbon and metal ions makes the transition metals in metal/carbon composites have higher dispersion, more active sites and faster charge transfer, thus enhancing the catalytic performance. Therefore, developing highly efficient electrolysis materials based on MOF-derived heteroatom-doped carbon matrix, incorporated with transition metals is significantly necessary for advancing the oxygen electrochemical process in ZABs [116].

#### MOF-Derived Transition Metal Doped Carbon Composites

**Fig. 4:**
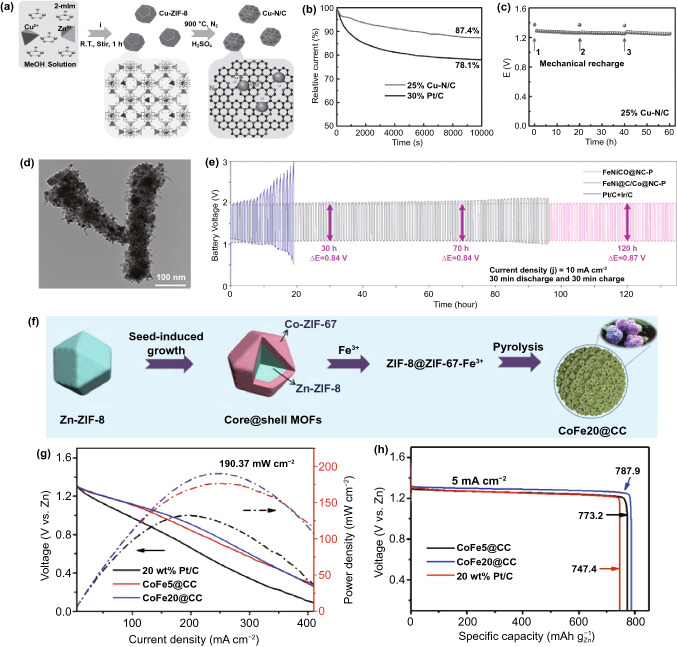
**a**–**c** Synthesis process, current–time response for ORR and ZABs performance of the Cu–N/C catalyst. Copyright © 2017 Wiley-VCH Verlag GmbH & Co. KGaA, Weinheim. **d** TEM image of FeNiCo@NC-P catalyst. Copyright © 2017 WILEY-VCH Verlag GmbH & Co. KGaA, Weinheim. **e** Cycling stability for the ZABs with FeNiCo@NC-P. Copyright © 2019 WILEY-VCH Verlag GmbH & Co. KGaA, Weinheim. **f**–**h** Synthesis diagram, power density plots and the specific capacities of forming open carbon cages embroidered spherical structure in ZABs. Copyright © 2019 WILEY-VCH Verlag GmbH & Co. KGaA, Weinheim

Incorporation of transition metal clusters (e.g., Fe, Co, Ni) into the carbon matrix has been demonstrated to be effective for oxygen electrocatalysis [117–119]. Owing to the diversity of MOFs, it is promising to synthesis efficient MOF-derived metal/carbon composites with controlled structure by selecting proper MOF precursor and post treatment process [120]. ZIFs, as molecular sieve polyhedral nanocrystals, are a kind of widely used template to synthesis Co incorporated nitrogen-containing carbon materials [121, 122]. In the presence of ZIF-67 filled silica nanoparticles and an additional high-temperature decomposable nitrogen source, a nitrogen-rich hollow carbon cage composite material containing Co nanoparticles (Co@NHCC) was synthesized [123]. When Co@NHCC-800 was used as a gas-permeable electrode of ZABs, its open-circuit voltage was as high as 1.49 V, and the discharge peak power density was as high as 248 mW cm^−2^. The charge and discharge voltage gap after 12 h of cycling only increased by 0.1 V. In parallel with cobalt element, Lai et al. [124] used a simple and effective Cu coordinated MOF strategy to prepare the novel copper-nitrogen-carbon electrocatalyst (Cu–N/C) by direct pyrolysis of Cu-doped ZIF-8 polyhedron in an inert atmosphere without a template or surfactant (Fig. 4a). As shown in Fig. 4b, 25% Cu–N/C has higher operating durability than 30 wt% Pt/C catalyst. By supplementing the zinc anode and electrolyte, the constructed ZABs could work for a long time. After three times of so-called mechanical charging (Fig. 4c), the output voltage remained extremely stable without a significant drop, suggesting the great advantages of MOF-derived Cu–N/C in replacing precious metal-based materials in ZABs and other related energy conversion devices.

Besides, owing to their similar topological structures and unit cell parameters of ZIF-67 and ZIF-8, several works explored the potential of using these MOF hybrids as a platform to prepare transition metal doped carbon composites [125–130]. For example, the Yin group [131] put forward the synthesis of MO-Co@N-doped carbon (M means metal Zn or Co) by the pyrolysis of Co-contained ZIF-67 and Zn-contained ZIF-8 hybrid MOFs. With the only Zn as precursors, the pyridinic N as an active sites for ORR formed on the surface, while, the addition of Co metal node, the OER catalytic active species Co–N_*x*_ and Co^3+^/Co^2+^ were generated. Also, the synergy between the Zn and Co could promote the growth of multi-walled carbon nanotubes at high temperatures (greater than 700 °C), which was beneficial to charge transfer. It is demonstrated that the optimized CoZn-NC-700 showed excellent ZABs performance better than the Pt/C and IrO_2_ hybrid batteries. Another work also explored the merits of such hybrid MOF-derived carbon nanotubes [132] by employing two-dimensional bimetallic leaf-like zeolite-based imidazolate skeletons (ZIF-L) as precursors to prepare carbon nanotubes decorated with Co nanoparticles (Co–N-CNTs). When used as dual-function oxygen electrocatalysts for the air electrode in ZABs, the primary batteries exhibited a high open-circuit potential of 1.365 V, a current density of about 90 mA cm^−2^, and a peak power density of about 101 mW cm^−2^. All these works evidenced the priority of ZIF-derived carbon nanotubes for ZABs in comparison to the commercial carbon nanotubes and Pt/C or IrO_2_, suggesting the MOFs.

Ni nanoclusters are also frequently used as catalytic active species for oxygen electrolysis as compared to Co, the addition of Ni would offer the catalysts optimized electron structure and thus enhanced electrochemical performance. By employing a multi-shelled two-dimensional NiCo-MOF@ZIF-L(Zn)@ZIF-67 composite, Co/Ni-doped porous carbon was successfully synthesized [133]. NiCo-NC as the air electrode catalyst in the ZABs shows a large specific capacity of 792.8 mAh g^−1^, a peak power density of 243.4 mW cm^−2^, a small charge and discharge voltage gap of 0.84 V at 20 mA cm^−2^, with good cycling stability. Thus, the design and construction of multi-shell 2D MOFs provide new opportunities for energy conversion and storage applications. Besides, trimetallic catalysts are also studied for oxygen catalysis for the potential synergetic effect [134]. Therefore, it is necessary to design dual- or multi-MOFs to achieve a good adjustment of porosity and increased intrinsic activity through the synergistic effect of multi-metal components. Chen et al. [61] proposed a new strategy for the preparation of multi-metal-based porous carbon nanorod composites by the combination of bifunctional MOFs (FeNiCo@NC-P, Fig. 4d). Compared with a single MOF-derived carbon material, N dopants and various metals such as Fe, Co and Ni were added to the prepared catalyst. At the same time, the micro-/mesoporous structure was successfully prepared using two MOF precursors, and the one-dimensional carbon nanorod structure was maintained during pyrolysis at high temperatures. All these characteristics ensure that FeNiCo@NC-P had highly active ORR and OER sites, fast charge transfer, reduced interface resistance, effective oxygen and electrolyte diffusion to show excellent dual-function electrocatalytic activity. In addition, the practical application of FeNiCo@NC-P in ZABs shows a low voltage gap and long-term durability of more than 130 h at 10 mA cm^−2^ (Fig. 4e), and its performance was better than commercial precious metal benchmarks. These works not only provide a competitive dual-function oxygen electrocatalyst for ZABs but also open up new ways to design and prepare MOF-derived metal cluster/carbon composite materials with adjustable catalytic performance.

#### MOF-Derived Alloy Doped Carbon Composites

The formation of alloy is another effective method to improve oxygen electrocatalytic activity. The excellent catalytic activity is attributed to the synergistic effect of various metal components, which provides a variety of well-defined active sites to promote the electrocatalytic reaction [135]. Bimetallic alloys such as CoNi, FeNi or FeCo combined with carbon materials have been considered to have higher activity and stability than their single-element counterparts. At the same time, the alloy particles can improve graphitization and promote electron transfer during carbonization, while carbon carrier can prevent acid corrosion, surface oxidation and agglomeration of alloy particles. MOFs have a variety of characteristics and can be transformed into alloy-carbon compounds in-situ under heat treatment, which has become an ideal precursor for designing electrocatalysts [136].

Recently, MOF-derived FeCo alloys have been extensively studied for oxygen electrocatalysis. By using amorphous ZIF-67 decorated amorphous fibers as precursors, Niu and Yang [137] firstly synthesized mesoporous nitrogen-doped graphene (MNG) compounds, then the MNG-CoFe prepared by decorating the MNG with graphene-coated CoFe alloy nanoparticles. As expected, the obtained MNG-CoFe assembled air electrodes in the ZABs showed high power density and cycle durability. In another work, the authors prepared nitrogen-doped carbon nanofibers coated with FeCo alloy nanoparticles by the pyrolysis of the CoFe-PBA@PAN precursor in argon atmosphere at 800 ℃ [138]. The peak power density of the ZABs based on FeCo-NCNFs-800 was as high as 74 mW cm^−2^, with the strong cycle stability upto 125 cycles for 42 h. They claimed that the 1D fibrous structure, FeCo alloy nanoparticles, extensive mesoporous structure and sufficient Co–N (pyridine-N) catalytic active sites were responsible for the high-performance ZABs. In parallel, by introducing guest iron ions into core–shell Zn@Co-MOFs precursor followed by in-situ pyrolysis, the open carbon cage self-assembled into a hydrangea-like three-dimensional superstructure connected by carbon nanotubes decorated with FeCo alloy nanoparticles (Fig. 4f) [139]. Surely, its excellent electrocatalytic performance was related to the unique superstructure, both conductive and porous channels to achieve rapid electron transfer and effective mass transfer, as well as abundant Co/Fe catalytic sites and significant synergistic effects. As an air electrode catalyst in the ZABs, it showed perfect performance, reaching a high peak power density of 190.3 mW cm^−2^, an ultra-high capacity of 787.9 mAh g^−1^ and an amazing energy density of 1012 Wh kg^−1^ (Fig. 4g, h).

Although MOF-derived alloy doped carbon materials show promising oxygen electrocatalytic performance in ZABs, design and synthesis is still in their infancy, facing different challenges. One of the big challenges is the side effects of aggregation and phase separation during the reduction process of the bimetallic phase under heat treatment. The second is the limited coordination of metal ions and organic ligands, which limits the choice of metal–ligand combinations [140, 141]. Therefore, more emerging strategies need to be developed to further discover MOF-derived alloy-carbon catalysts with unique catalytic properties. Meanwhile, the suitable metal precursors with the required proportion and composition can be selected by the prediction of the phase diagram and enthalpy of formation and can coordinate with MOF ligands through different reactions such as gas-phase, liquid-phase and solid-phase [135].

### MOF-Derived Metal Compound/Carbon Composites

For the past few years, transition metal compounds have long been studied as oxygen electrocatalysts, but the inherent low conductivity and poor dispersion of nanoparticles seriously hinder their electrocatalytic activity. Consequently, transition metal compound combined with carbon materials, MOF-derived carbon materials particularly, can effectively overcome the above concerns [142–144]. By far, various MOF-derived metal compound/carbon composites have been reported for oxygen electrocatalysis, which mainly includes metal oxides, metal sulfides/carbides/phosphides and other metal compounds composite carbon materials.

#### Metal Oxides

Transition metal oxides are the most widely studied compounds for oxygen electrolysis. On the one hand, they are easy to prepare in oxidizing or alkaline environments, on the other hand, they are very stable during the oxygen electrocatalytic process due to the oxidized surface composition. When MOFs employed as precursors, oxygen-rich vacant metal oxides can be obtained, which would offer much higher ORR/OER activity than ordinary materials. Among various MOFs, Co containing MOFs are mostly studied. These MOF-derived cobalt oxide/carbon composite material can help to improve the conductivity and dispersibility of the cobalt oxide, thereby exposing the intrinsic active sites in the catalyst [145]. Compared with pure Co_3_O_4_, the MOF-derived hybrid carbon material based on Co_3_O_4_ doped with nitrogen, sulfur, and phosphorus showed better electrochemical activity [146–152]. For instance, Ren et al. [153] prepared a porous nanowire array composed of Co_3_O_4_ nanoparticles and carbon species by carbonizing ZIF-67 directly grown on nickel foam. The resulting hybrid material was used as an air catalyst for ZABs, which exhibited a large peak power density of 118 mW cm^−2^ and promising operating stability.

**Fig. 5:**
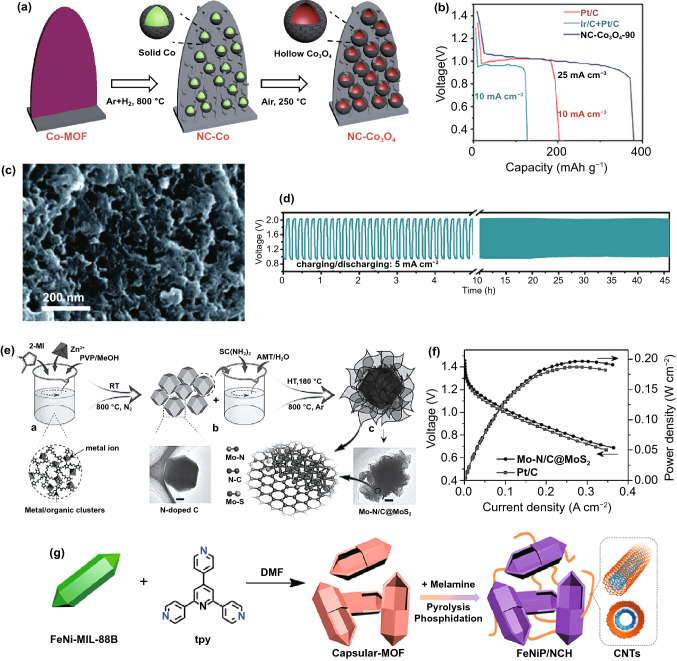
**a** Fabrication process and **b** voltage-capacity curves (solid-state Zn–air batteries) for hierarchical NC-Co_3_O_4_ arrays on flexible carbon cloth. Copyright © 2017 WILEY-VCH Verlag GmbH & Co. KGaA, Weinheim. **c** SEM image and **d** long-term charging-discharging cycling tests of Co_9_S_8_@TDC-900 catalyst. Copyright © 2019 The Royal Society of Chemistry. **e** Schematics for the synthesis and **f** V-I polarization and power density curves of Mo–N/C@MoS_2_ catalyst. Copyright © 2017 WILEY-VCH Verlag GmbH & Co. KGaA, Weinheim. **g** Preparation process diagram of FeNiP/NCH catalyst. Copyright © 2019 American Chemical Society

Designing unique nanostructures also play a significant role in the improvement of oxygen catalytic activity. By using a well-designed MOFs precursor, a N-doped carbon nanowall array embedded with irregular hollow Co_3_O_4_ nanospheres electrocatalyst (NC-Co_3_O_4_) was designed [154]. The authors explained that the surface of the metal nanoparticles covered with a layer of graphite onion during the carbonization process (Fig. 5a). Then the carbon onion-coated Co nanoparticles inhibited the Kirkendall effect at the nanoscale, promoted the formation of irregular hollow Co_3_O_4_ nanospheres, and offered them pleasurable catalytic properties for OER and ORR. Furthermore, the integrated NC-Co_3_O_4_/CC was directly used as an additive-free air cathode for flexible all-solid ZABs, showing a large capacity of 387.2 mAh g^−1^ (Fig. 5b), exceptional cycle stability and mechanical flexibility, which was significantly better than Pt-based and infrared ZABs. In another work, by a facile carbonization-oxidation method, the 3D-on-2D MOF precursor on carbon cloth (ZIF-L-D/CC) was converted to nitrogen-doped carbon and Co_3_O_4_ nanoparticles (ZIF-L-D-Co_3_O_4_/CC) [155]. This unique layered MOF on MOF (3D-on-2D) structure undoubtedly promoted the reaction kinetics and proton transport. Meanwhile, the tight protection of Co_3_O_4_ nanoparticles by N-doped carbon also provided prominent electrochemical activity and stability. In particular, when the catalyst was applied to air cathode inflexible all-solid-state ZABs, it exhibited high open-circuit potential (1.461 V), capacity (815 mAh g^−1^ at 1 mA cm^−2^), energy density (1010 Wh kg^−1^), exceptional cycling stability as well as outstanding mechanical flexibility. This work could push the enormous development of new-type flexible energy conversion and storage devices.

In addition to cobalt oxide, manganese oxide is another promising oxygen electrocatalytic materials for its variable geometry. To resolve the low conductivity of single manganese oxide, Chen et al. [156] selected ZIF-67 supported ultra-thin MnO_2_ hollow nanowire as a precursor, MnO@Co–N/C nanomaterials with controllable diameter were obtained after pyrolysis. The synergistic effect between the MnO and porous Co–N/C provide excellent catalytic activity and performance of the ZABs, even better than Pt/C and RuO_2_ mixed catalysts. To further investigate such MOF-derived MnO/Co for oxygen electrolysis, a heterogeneous MnO/Co interface in a porous graphite carbon polyhedron (MnO/Co/PGC) was prepared by using a bimetallic metal–organic framework as a precursor [157]. In-situ generated Co nanocrystals could not only form a highly conductive heterointerface, overcome the disadvantage of poor OER activity, but also promote the formation of graphitic carbon. Therefore, MnO/Co/PGC shows excellent activity and stability for both OER and ORR. Importantly, domestic ZABs exhibited outstanding performance, including peak power density of 172 mW cm^−2^, specific capacity of 872 mAh g^−1^, as well as excellent cycle stability, better than commercial Pt/C mixed catalyst with RuO_2_. Different from the simple mixture of MnO_2_ with MOF to prepare metal oxides/carbon composite, this work emphasizes the synergy of heterogeneous interfaces in oxygen electrocatalysis, thereby providing a promising way to develop advanced zinc-air cathode materials.

#### Metal Sulfides/Phosphides/Carbides

MOF-derived carbon-based transition metal sulfides/phosphides/carbides composites are also hotspots in ZABs [158]. For instance, Co/Co_*x*_S_*y*_@S, N-doped carbon fibers were prepared by Co-MOFs fibers under hydrothermal conditions followed with pyrolysis at 800 °C [159]. The catalyst showed excellent bifunctional ORR and OER activity, as well as high power density and favorable durability in rechargeable ZABs. To further investigate such composite on the oxygen electrolysis, N, O and S-doped carbon matrix embedded with Co_9_S_8_ nanoparticles (Co_9_S_8_@TDC, Fig. 5c) was produced by direct carbonization of a novel Co-MOF constructed by SPDP (4,4′-(sulfonylbis(4,1-phenylene))dipyridine) and H_2_BDC (1,4-benzene dicarboxylic acid) ligands [160]. The inherent activity of Co_9_S_8_ nanoparticles and the heteroatom-doped carbon shell promote the catalytic performance of OER and ORR. The Co_9_S_8_@TDC-900 was used as an air cathode catalyst layer in rechargeable ZABs, which provided considerable open-circuit voltage of 1.50 V and long-term charge–discharge stability (Fig. 5d). In addition to the cobalt sulfide, the study on molybdenum sulfide is also quite remarkable toward ORR electrocatalysis. Considering the outstanding flexibility, ultra-high surface area, layered pore structure and high catalytic activity of MOFs and metal dihydrogen disulfide, Mu’s team reported a highly efficient electrocatalyst based on a vertically aligned MoS_2_ nanosheet hierarchically interconnected Mo–N/C frame produced by carbonization of ZIF-8 resulting in the formation of an interface Mo–N coupling center (Fig. 5e) [161]. Surprisingly, when used as a cathode electrocatalyst in ZABs, it showed a high power density of about 196.4 mW cm^−2^ (Fig. 5f), and the voltammetry efficiency at 5 mA cm^−2^ was about 63%. Even after 48 h at 25 mA cm^−2^, it still had excellent cycle stability. Such perfect electrocatalytic performance was attributed to the synergistic effect of unique chemical composition, unique three-phase active site and layered pore frame for rapid mass transfer.

Owing to the similar catalytic properties of phosphides with sulfides, there are also a few studies on MOF-derived metal phosphides/carbon composites [162, 163]. In comparison to transition metal sulfides-based oxygen electrocatalysts, phosphides display enhanced catalytic activity but limited stability, while MOFs just provide a potential solution for such issue. For example, based on the pyrolysis of ZIF-67 and dicyandiamide, Hao et al. [164] synthesized carbon polyhedral penetrating bamboo-like carbon nanotubes by a three-step chemical method. This polyhedron provided large interfacial areas for catalytic reactions. The compositions of Co, CoP and hairy N-doped carbon in the catalyst made it possess higher catalytic activity. As an air cathode in the rechargeable ZABs, it still had high round-trip efficiency, low overpotential and stable voltage platform after 100 cycles. In another important work, the authors used MOF enveloped protein and melamine as starting materials, then a N-doped envelope carbon-based framework was prepared by the pyrolysis-phosphine reaction with the iron-nickel phosphide nanoparticles fixed to the envelope connected by a large number of carbon nanotubes on carbon (Fig. 5g) [165]. The synergistic effect between the carbon skeleton and the highly surface-exposed phosphide sites made the material exhibit highly efficient multi-functional electrocatalysis in HER, OER and ORR. It was a qualified component for rechargeable ZABs with the peak value power density was 250 mW cm^−2^, and perfect stability was up to 500 h.

Apart from the transition metal sulfides/phosphides, MOF-derived carbide/carbon composites also show good electrocatalytic properties [166]. By assembling ZIF-8 nanoparticles (NPs) within the polyacrylonitrile nanofibers with an electrospinning method, Liu et al. [167] reported Fe_3_C NPs embedded in Fe–N-doped porous carbon nanofibers as a bifunctional oxygen electrocatalyst applied in ZABs. Similarly, by using ZIF-8 as a precursor and uniformly distributed ultrafine α-MoC nanoparticles as a model electrocatalyst, a nitrogen-doped layered porous carbon material (α-MoC/NHPC) was prepared [168]. Theoretical studies have shown that α-MoC on NHPC could effectively reduce the energy barrier for proton generation during the hydrolysis process, and ultimately promote the proton coupling ORR dynamics. The α-MoC/NHPC catalyst synthesized with the assistance of NaCl had the advantages of ultrafine nanoparticles and MOF-derived layered porous carbon structure, it exhibited excellent ORR performance with a half-wave potential as high as 0.88 V. As the air electrode of the ZABs, its peak power density was 200.3 mW cm^−2^, which had long-term stability.

#### Other Compounds

**Fig. 6:**
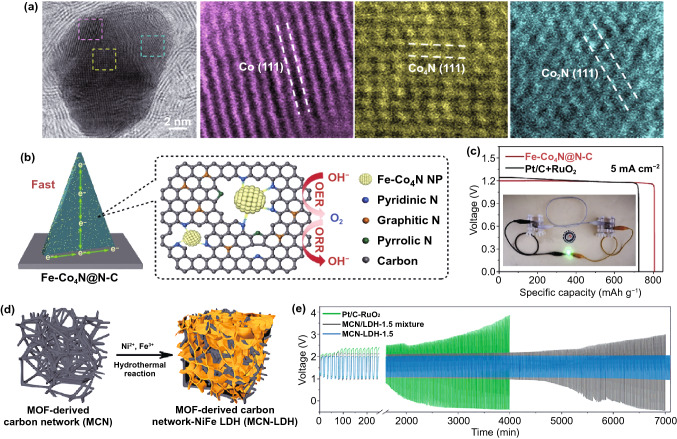
**a** High-magnification STEM ABF image of NC-Co/CoN_*x*_ catalyst and the lattice fringe from different parts of it. Copyright © 2018 Elsevier B.V. **b** Schematic synthesis process and **c** the specific discharging capacities curves of the Fe–Co_4_N@N–C for air electrode. Copyright © 2019 Elsevier B.V. **d** Preparation diagram and **e** continuous discharge/recharge voltage records of air cathodes in zinc-air catalyzed by of MOF-derived carbon network-NiFe-LDH catalyst. Copyright © 2019 American Chemical Society

N-doping and nitrides have been proved to be active species for oxygen electrolysis by promoting the charge transfer in the catalytic process [169]. Because the pyrolysis of MOFs may lead to the loss of nitrogen content, it is feasible to enrich the amount of nitrogen in MOF derivatives by nitriding [170]. For instance, Guan et al.[171] demonstrated the design of NC embedded with Co/CoN_x_ nanoparticles (NC-Co/CoN_*x*_) by carbonizing Co-ZIF-L followed with nitriding process (Fig. 6a), which provided abundant active sites, high-density interfaces and short ion diffusion paths. This highly integrated electrode combines the advantages of each component, showing high electrochemical performance and strong mechanical stability. A MOF-derived metal nitrides/N-doped carbon composites (Fe–Co_4_N@N–C) with richly accessible pyridine-N-M active sites were developed by ammonization [172]. Owing to the strong coordination between metal center and pyridinic nitrogen, Fe doping in Co-MOF promoted the formation of a large number of pyridine-N-M active sites for ORR (Fig. 6b). Therefore, when used as the air cathode in liquid Zn-air batteries, the electrocatalyst achieved a high specific capacity of 806 mAh g^−1^ at 5 mA cm^−2^ (Fig. 6c) and excellent cycle stability.

Furthermore, MOF-derived metal selenides/carbon composites have also attracted enormous attention toward oxygen electrocatalysis. By direct selenization of ZIF-67, the ultrafine Co_0.85_Se nanocrystals coupled with N-doped carbon was developed [173]. In-situ carbonization of ZIF-67 could ensure a smaller size of Co_0.85_Se nanocrystal to increase the active sites to obtain a higher electrocatalytic activity. Also, the Co_0.85_Se@NC catalyst inherited the unique porous structure of the ZIF-67 template, which could increase its surface area in contact with electrolyte, thus effectively promoting the transfer of electrolyte and the rapid diffusion of gas products for better performance. The catalyst assembled in ZABs played a very low charge–discharge voltage gap of 0.80 V and a life span of 180 cycles at 10 mA cm^−2^. Because the electron transfer of metal compound in the catalytic process can affect the intrinsic conductivity of the catalyst. Therefore, with the assistance of MOFs, both phosphorous and selenium were introduced during pyrolysis to form a complex catalyst CoFe–P–Se/NC [174]. A low voltage gap of 0.719 V at 200 mA cm^−2^, the high power density of 104 mW cm^−2^, and the high energy density of 805 Wh kg^−1^ were achieved when used in a rechargeable battery.

As a typical two-dimensional material, layered double hydroxides (LDHs) have long been studied for oxygen electrochemical reactions, especially the OER process, but the applications of MOF-derived hydroxides in ZABs are inadequate [175]. Recently, Qian and his colleagues explored the performance of the NiFe layered double hydroxides for ZABs [176]. They prepared the OER active NiFe-LDH composites supported on ORR active MOF-derived carbon network (MCN) as Janus-ORR/OER electrocatalyst for the rechargeable ZABs. Through simple hydrothermal reaction, LDH nanoplates could be easily fixed on MCN (Fig. 6d). The interaction between MCN and LDH enhanced the OER activity, but ORR activity remained positive. When MCN-LDH was further installed in the rechargeable ZABs, it could operate continuously for more than 100 h at 10 mA cm^−2^ without significant performance loss (Fig. 6e). The life of the batteries was tripled as compared to the standard precious metal catalyst Pt/C-RuO_2_ assembled batteries. Therefore, the MOF-derived hydroxides cases show great potential in the development of the ZABs and other related electrochemical energy storage systems [177].

## Conclusion and Outlook

**Fig. 7:**
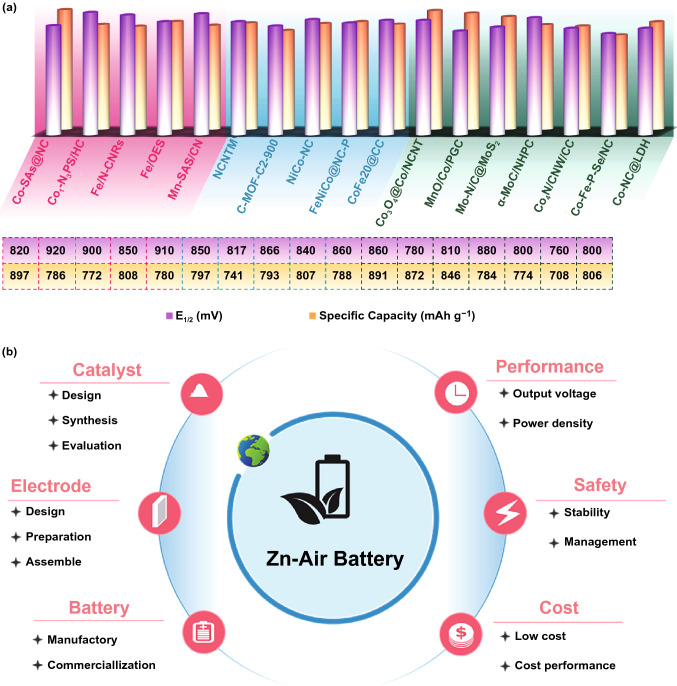
**a** Summary of the half-wave potential (*E*_1/2_) and specific capacity for advanced MOF-derived oxygen electrocatalysts for ZABs. **b** Proposed aspects for the future development of ZABs

With the increasing demand for energy conversion devices in modern society, air electrode is of immense significance for ZABs. For the improved performance and durability of ZABs, the exploration of efficient oxygen electrocatalysts is urgent and meaningful. MOFs featured with diverse inorganic metal nodes and organic ligands provide an abundant platform for the design of low cost and highly efficient oxygen electrocatalysts for the air cathode in ZABs. In this review, with a brief introduction of the fundamentals on oxygen electrolysis in ZABs, the recent advances on MOF-derived non-noble metal–oxygen electrocatalysts are successively reviewed for ORR and OER from the category of metal-free carbon materials, single-atom catalysts, metal/carbon composites and metal compound/carbon composites (Table 1). In particular, the structure-performance relationship of these MOF-derived non-noble metal oxygen electrocatalysts for zinc-air batteries (Fig. 7a) in term of their oxygen electrocatalytic activity and specific capacity. Despite great achievements have been made in the field of MOF-derived oxygen materials for zinc-air batteries, they still face a host of numerous challenges.

**Table 1:** Summary of the recent MOF-derived oxygen electrocatalysts reported in the literature

Derivatives	Precursor MOFs	ORR/OER electrolyte	ORR: *E*_onset_ /*E*_1/2_ (V vs. RHE)	OER: E_j=10_ (V vs RHE) /Overpotential (mV)	ΔE = E_j=10_ – E_1/2_ (V)	Open-Circuit Potential (V)	Peak Power Density (mW cm^−2^)	Specific Capacity (mAh g^−1^) @Current Density (mA cm^−2^)	Cycle Stability Time (h) @ Current Density (mA cm^−2^)	Refs
*MOF-derived metal-free carbon–oxygen electrocatalysts*										
SCNS	ZIF-8@PVP	0.1 M KOH –	0.85/0.77	–	–	1.47	163	–	> 170@5	[71]
NHPC-900–1000	ZIF-8	0.1 M KOH –	–/0.84	–	–	–	–	–	–	[78]
BHPC-950	SiO_2_@ZIF-8	0.1 M KOH –	0.93/0.81	–	–	1.44	197	797@20	180@20	[80]
HNCSs	ZnO@ZIF-8	0.1 M KOH –	0.92/0.82	–	–	–	–	–	–	[81]
Carbon-L	ZIF-7	0.1 M KOH –	0.861/0.679	–	–	–	–	–	–	[82]
BNPC-1100	MC-BIF-1S	0.1 M KOH 6.0 M KOH	0.894/0.793	1.55/–	0.757	–	–	–	100@2	[85]
*MOF-derived single-atom oxygen electrocatalysts*										
NC-CoSA	Co-based MOF	1.0 M KOH 1.0 M KOH	1.00/0.87	–/360	0.72	1.411	20.9	–	~ 41.7@10	[103]
CoSA@NCF/CNF	Co-ZIF-Ls	0.1 M KOH 1.0 M KOH	–/0.88	1.63/400	0.75	1.41	–	530.2@6.25	> 15@6.25	[104]
CoSA + Co_9_S_8_/HCNT	ZIF-67	0.1 M KOH 0.1 M KOH	0.920/0.855	1.56/–	0.705	1.45	177.33	788@100	24@5	[105]
Fe-SAs/NPS-HC	ZIF-8	0.1 M KOH –	–/0.912	–	–	1.45	195	–	~ 55.6@5	[106]
FeN_*x*_-embedded PNC	ZIF-8	0.1 M KOH 0.1 M KOH	0.997/0.86	–/390	0.775	1.55	278	–	55@5	[109]
Fe–N_*x*_–C	Fe-Phen@ZIF-8	0.1 M KOH 0.1 M KOH	1.05/0.91	–/600	0.92	1.51	96.4	641@10	300@5	[111]
Fe/OES	Fe/ZIF-8@SiO_x_	0.1 M KOH –	1.00/0.85	–	–	1.51	186.8	807.5@5	> 130@5	[112]
Mn-SAS/CN	Mn(CH_3_COO)_2_@ZIF–8	0.1 M KOH –	1.00/0.91	–	–	–	226	780@10	25@10	[114]
MOF-derived metal cluster/carbon composite oxygen electrocatalysts										
Co-MOF-800	ZIF-8	0.1 M KOH 0.1 M KOH	–/0.84	1.68/-	0.84	1.38	144	671.6@10	85@1	[19]
NC@Co-NGC DSNCs	ZIF-8@ZIF-67	0.1 M KOH 0.1 M KOH	0.92/0.82	1.64/-	0.82	1.45	109	617@2	56@10	[117]
Co@NHCC-800	ZIF-67	0.1 M KOH 1.0 M KOH	0.938/0.837	1.512/-	0.675	1.49	248	-	12@10	[123]
CoNi-MOF/rGO	CoNi-MOF	0.1 M KOH 1.0 M KOH	0.88/0.718	-/318	0.83	1.37	97	711@5	120@5	[125]
ZIF-9_Fe3_Pyrol	ZIF-9	0.1 M KOH 0.1 M KOH	0.9/0.74	1.55/-	0.81	-	32	815@5	15@2	[128]
CoZn-NC-700	Zn(mim)_2_·(Hmim)_1/2_·(H_2_O)_3/2_	0.1 M KOH 0.1 M KOH	0.98/0.84	-/390	0.78	1.42	152	578@10	∼64.2@10	[131]
Co–N-CNTs	Co/Zn(1:1) ZIF-L	0.1 M KOH 0.1 M KOH	0.97/0.9	1.69/-	0.79	1.365	101	-	15@2	[132]
Ni_3_Fe/Co–N-C	Co,ZIF-8	0.1 M KOH 0.1 M KOH	–/0.79	1.54/310	0.75	1.39	68	-	65@10	[134]
MNG-CoFe	ZIF-67	0.1 M KOH 0.1 M KOH	0.98/–	1.62/390	0.64	∼1.5	~ 97.7	-	18@10	[137]
1.5FeNi@NCNT	1.5Fe/Zn-ZIF @PDA-Ni	0.1 M KOH 1.0 M KOH	0.95/0.86	-/230	0.60	1.44	114	-	12@5	[140]
*MOF-derived transitional metal compound/carbon composite oxygen electrocatalysts*										
Co@Co_3_O_4_@NC-900	ZIF-67	0.1 M KOH 1.0 M KOH	–/0.8	1.6/-	0.8	-	64	685@5	~ 80@5	[148]
NC-Co_3_O_4_-90	Co-MOF	1.0 M KOH 1.0 M KOH	0.91/0.87	-/358	0.718	1.44	82	387.2@25	100@10	[154]
ZIF-L-D-Co_3_O_4_/CC	ZIF-L-D	1.0 M KOH 1.0 M KOH	0.97/0.90	-/310	0.64	1.660	75	852@5	~ 384@5	[155]
MnO@Co–N/C	ZIF-67	0.1 M KOH 0.1 M KOH	–/0.83	1.76/-	0.93	1.445	130.3	-	633@5	[156]
Co_9_S_8_@TDC-900	[Co(BDC)_2_(SPDP)_2_(DMF)(H_2_O)]	0.1 M KOH 1.0 M KOH	–/0.78	1.56/330	0.78	1.5	101.5	-	~ 45@5	[160]
FeNiP/NCH	FeNi-MIL-88B	0.1 M KOH 1.0 M KOH	–/0.75	1.59/250	0.84	1.48	250	-	500@10	[165]
Fe_3_C@FeN@CNF-2	ZIF-8	0.1 M KOH 0.1 M KOH	0.94/0.84	1.67/-	0.83	1.44	-	640@10	> 50@10	[167]
NC-Co/CoN_*x*_	Co-ZIF-L	1.0 M KOH 1.0 M KOH	0.928/0.878	-/289	0.649	1.40	41.5	-	> 400@10	[171]
Co_0.85_Se@NC	ZIF-67	0.1 M KOH 1.0 M KOH	0.912/0.817	1.55/-	0.733	~ 1.4	268	-	30@10	[173]
Co-NC@LDH	ZIF-L	0.1 M KOH 0.1 M KOH	–/0.800	-/389	0.819	1.41	107.8	806@5	300@5	[177]

Here, we put forward several criteria for the commercialization of ZABs in future from the design of catalysts, electrode and batteries by considering the performance, safety and cost (Fig. 7b). Design and construction of efficient electrode materials are always of uppermost priority, the structural diversity of MOFs makes the materials system very complex, selection of proper MOF platform is highly critical for design MOF-derived oxygen electrocatalytic materials. Using high throughput technology, one can calculate and predict the possibility of the different MOF compositions and structures on the performance of final products, and thus accelerating the manufacturing speed of MOF-derived air electrode materials [178, 179]. Besides, the direct applications of MOFs electrocatalysts in ZABs are rarely studied, the ORR catalytic performance of pure MOFs has not reached the point of direct applications, with the aid of high throughput screening, the fast selection of active metal and organic ligands becomes impossible and accelerated for the design of catalytic active MOFs for ORR.

At present, the accurate structure elucidation of as-prepared MOFs derivatives is particularly useful for studying their structure–activity relationship during oxygen electrocatalysis in ZABs. Currently, growing individuals choose in-situ testing technology to explain the process and mechanism of OER, ORR and other electrocatalysis [180–182]. By the combination of electrode and batteries test with advanced in-situ synchrotron radiation technology, it would appreciate to fully characterize the structural evolution of the catalysts during the electrochemical reactions and batteries service. Over the past decade, intense research activities have been made for the oxygen electrocatalysts and air cathode, and a shift of research attention from air cathodes to Zn anodes would also advance the current ZABs.

In addition to the aforementioned challenges, the commercialization of current ZABs requires further balance in performance, safety and cost. The improvement of performance, such as power density, energy density and cyclability, could be achieved by optimizing the electrode materials, batteries configuration, electrolytes and operation conditions. For example, developing MOFs derivatives with enhanced graphitization and open-framework or 2D intercalation MOF electrode with tailored architecture; improve the utilization of electrolyte and prevent leakage; widen the output voltage by controlling the overpotential. Apart from high performance, safety is always the key aspect of any future battery technology in our daily life, especially for ZABs in comparison to current Li-ion batteries. Also, low cost, determining the large-scale application of ZABs, is crucial, which can be realized by using earth-abundant resources, simple manufacturing processes and facile control systems. Therefore, achieving the balance of cost and performance is the main task for the fast-growing research on ZABs in future.

While commercialization progress of ZABs requires more and more research work, it is believed that the progress in electrode materials innovations will boost the performance of ZABs in the coming years. At the same time, the structure of ZABs, the electrolyte and other aspects are all need to be improved so that the performance of ZABs could be further advanced in future. By considering the balance of high performance, safety and low cost, future developments in this area will surely advance the large-scale application of high-performance ZABs.
